# Acknowledgement to Reviewers of *Nutrients* in 2017

**DOI:** 10.3390/nu10010099

**Published:** 2018-01-16

**Authors:** Nutrients Editorial Office

**Affiliations:** MDPI AG, St. Alban-Anlage 66, 4052 Basel, Switzerland

Peer review is an essential part in the publication process, ensuring that *Nutrients* maintains high quality standards for its published papers. In 2017, a total of 1340 papers were published in the journal. Thanks to the cooperation of our reviewers, the median time to first decision was 21 days and the median time to publication was 53 days. The editors would like to express their sincere gratitude to the following reviewers for their time and dedication in 2017:

Aagaard, Niels KristianLivesey, GeoffreyAarabi, MahmoudLivolti, GiovanniAaron, Jean E.Lo Monte, Attilio IgnazioAbe, SumikoLo Scalzo, RobertoAbedelmalek, SalmaLocatelli, FrancescoAbenavoli, LudovicoLocatelli, MarcelloAbi Nader, PatrickLöfblom, JohnAbkowitz, Janis L.Loiselle, Denis S.Aboud, FrancesLøken, Elin B. Abraham, Bincy P.Lombardi, AngelaAbraham, ChristieLombardo, CaterinaAbrahamsson, ThomasLonardo, AmedeoAbt, Michael C.London, EdraAceña, ÁlvaroLoor, Juan J.Acuña-castroviejo, DarioLopez Jornet, PiaAdamkova, AnnaLópez, Luis CarlosAdegboye, AmandaLopez, NanetteAdekanmbi, Victor T.Lopez-Escamez, Jose A.Adhami, Vaqar M.Lopez-Miranda, JoseAdriouch, SoliaLópez-Sobaler, Ana M.Aerenhouts, DirkLopez-Valcarcel, Beatriz GonzalezAfrin, SadiaLorenzo, ÓscarAggarwal, AbhishekLövestam, ElinAggarwal, Bharat B.Lovreglio, PieroÁgh, TamásLow, DorrainAghajafari, FaribaLowe, NicolaAgil, AhmadLu, Louise WeiweiAgostoni, CarloLubahn, DennisAheto, Justice Moses K.Lucey, AliceAhluwalia, Amrita Ludy, Mary-JonAhmet, IsmayilLue, Ko-HuangAires, AlfredoLuick, Bret R.Alaedini, ArminLukaszuk, JudithAlaofe, HalimatouLund, Elizabeth K.Albonici, LoredanaLund, JennyAlbrecht, Sandra S.Lundberg, JonAldawsari, FahadLundin, KnutAl-Dujaili, EmadLung, Ming-YeouAlexy, UteLuo, JinAl-Hamdani, MohammedLupiáñez, José AntonioAllegaert, KarelLutfi Royo, EsmailAllen, AndrewLutjohann, DieterAlmajano, María PilarLutsey, PamAl-Nakkash, LaylaLutz, ThomasAlonso-Magdalena, PalomaLuzza, FrancescoAlsharairi, NaserLy, Sun YungAltamura, SandroLykkesfeldt, JensAltieri, BarbaraLynch, Heidi M.Aluko, RotimiLyons, Paul E.Álvarez, CarlosLysne, VegardAlwahsh, Salamah M.M.Fuller, PatrickAmbigapathy, GaneshMa, DavidAmedei, AmedeoMa, WenxinAmoaku, Winfried M.Ma, YunshengAmodio, PieroMa, Zheng FeeiAnadón, ArturoMacFarlane, Amanda J.Ananieva, ElitsaMachetti, FabrizioAnastasia, VlasovaMacia, LaurenceAndersen, Barbara VadMack, IsabelleAndersen, Catherine J.MacPherson, RebeccaAnderson, CherylMadeddu, RobertoAnderson, PeterMaeda, AkikoAndreas, NicholasMaehre, Hanne K.Aneskievich, Brian J.Maestro, MiguelAnestis, DougkasMaffeis, ClaudioAngadi, SiddharthaMafra, DeniseAngeles Martin, MariaMager, DianaAngelino, DonatoMaggi, DavideAngelucci, AdrianoMaggini, SilviaAnnibale, BrunoMahajna, JamalAnnunziata, AzzurraMaher, PamelaAnsari, RaisMahimainathan, LeninAnthias, ChloeMahoney, Sara E.Anthimopoulos, MariosMai, VolkerAnthony, TracyMaiorino, Maria IdaAntoine-Jonville, SophieMaki, KevinAntonakou, AngelikiMalavolta, MarcoAntonio, JoseMalemud, Charles J.Anvari, SaraMalik, Rayaz A.Aoi, WataruMalin, Steven K.Appleton, KatherineMallard, Simonette R.Araújo, João RicardoMallo, FedericoArboleya, SilviaMandò, ChiaraArcher, EdwardManganaris, GeorgeArcidiacono, BiagioManiam, JayanthiArciero, PaulManni, AndreaArdenghi, DiegoManninger, MartinArdévol, AnnaManoli, IriniArfuso, FrankMansueto, PasqualeAriño, AgustínManthou, EiriniAriyoshi, NobuhiroMantzioris, EvangelineArjmandi, BahramManuelli, MatteoArmand, MartineMarakis, GeorgiosArmanini, DecioMarangoni, FrancaArmstrong, Gavin R.Marcadenti, AlineArrick, DeniseMarchesini, GiulioArteaga, OlatzMarchini, CristinaArulselvan, PalasamyMaret, WolfgangArús, CarlesMargaritelis, NikosAsahina, KinjiMargerison, ClaireAsano, ShinichiMaría, Tabernero UrbietaAschbrenner, KellyMarigliano, MarcoAshcroft, FrancesMarín, MercedesAshman, AmyMariño, ElianaAspalter, RosaMarino, JosephAspatwar, AshokMariotti, FrançoisAterini, StefanoMarkaki, AnastasiaAtkins, Janice L.Markey, OonaghAtkinson, StephanieMarkhus, Maria WikAttanasio, ChiaraMarkou, Kostas. B.Atteritano, MarcoMarks, JoanneAttuquayefio, TukiMarlicz, WojciechAuguet, TeresaMarlow, GarethAugustin, HannaMarr, RobertAugustin, LiviaMarra, MaurizioAune, DagfinnMarrs, TomAuricchio, SalvatoreMarshall, SkyeAustin, GregoryMarshall, Teresa A.Avery, AmandaMarteau, PhilippeAvignone-Rossa, ClaudioMartel, FátimaAxelsson, JohnMartin, CamiliaAyonrinde, OyekoyaMartin, FinianAyton, ScottMartin, François-Pierre J.Azarpazhooh, ElhamMartín, FranzAzcárate-Peril, M. AndreaMartin, KristyAzcona, María CristinaMartín-Antonio, BeatrizAzmi, Asfar S.Martinelli, PasqualeAzzout-Marniche, DalilaMartínez, HomeroBaar, KeithMartinez, JessicaBaba, HideoMartinez, KristinaBach, ChristopherMartínez, RosarioBaecker, NatalieMartinez, VicenteBaenas, NievesMartinez-Castelao, AlbertoBagüés, AnaMartini, ClaudiaBahwere, PalukuMartini, DanielaBailey, LynnMartín-Orúe, Susana M.Bailey, ReganMartins, Ian JamesBains, PushpinderMartins, NatáliaBaïz, NourMartorana, AlessandroBajka, BalazsMaruyama, KeiBakardjian, HovagimMarventano, StefanoBaker, JulienMarverti, GaetanoBakillah, AhmedMarx, WolfgangBakker, BarbaraMarzullo, PaoloBaksh, ShairazMascherini, GabrieleBalantekin, KatherineMasilamani, MadhanBalcerczyk, AnetaMaslin, KateBaldassarre, Maria ElisabettaMaslova, EkaterinaBaldofski, SabrinaMassot, MalenBallmann, ChristopherMastrocola, RaffaellaBallocca, FlaviaMasuelli, LauraBally, LiaMatarese, Laura E.Bana, JinanMateu De Antonio, JavierBanach, MaciejMatheson, JesseBanerjee, AditiMathews, SureshBanerjee, Hirendra NathMatias, CatarinaBanerjee, KalpitaMatsubara, KeiichiBao, YongpingMatsumoro, KenjiBapputty, ReenaMatsumoto, HiroyukiBaragetti, AndreaMatsuura, BunzoBaranovski, TomMatsuzaki, KeiichiBarbaresko, JanettMatthys, ChristopheBarbul, AdrianMattijssen, FritsBarchetta, IlariaMattioli, Anna VittoriaBärebring, LinneaMattner, JochenBarffour, MaxwellMaughan, Ronald J.Barker, Edward D.Maukonen, MirkkaBarker, TylerMauriz, José L.Barnard, NealMavian, CarlaBarrea, LuigiMavrogonatou, EleniBarreira, João C. M.May, FelicityBarrero, AnnaMazoit, Jean-XavierBartelt, AlexanderMc Naughton, Lars R.Barvencik, FlorianMcCaffrey, TracyBaskaran, CharumathiMcCarthy, Sinéad N.Bassareo, Pier PaoloMcCaskill, MichaelBasso, KariMcClain, Craig J.Bates, ChristopherMcConnell, GlennBaudry, JuliaMcCourt, PeterBauer, JudithMcDougall, GordonBaum, JamieMcGill, Anne-TheaBaur, Daniel A.McKenna, Malachi J.Baute, VanessaMcLean, Rachael M.Bayat, VafaMcMahon, EmmaBazzano, AlessandraMcMahon, NickBeasley, SheaMcmillan, Donald C.Beberashvili, IliaMcmorrow, TaraBeck, KathrynMcNally, DayreBeckett, EmmaMcNeil, DavidBedard, AnnabelleMeacham, Susan L.Bednářová, MartinaMedler, Kathryn F.Beeken, RebeccaMeilhac, OlivierBeezhold, BonnieMejía-Benítez, AuroraBelay, BrookMelby, ChrisBell, ColinMellor, Duane D.Bell, DavidMellott, TiffanyBell, Jack J.Menaa, FaridBell, KirstineMendes, RosaBellisle, FranceMéndez, LucíaBello, Nicholas T.Méndez-Álvarez, EstefaníaBelury, MarthaMendonça, NunoBenatar, JocelyneMeng, HongdaoBendik, IgorMeng, Yuan XiangBenedec, DanielaMenkhorst, EllenBenn, Christine StabellMercadel, LucileBentel, Jacqueline M.Mercado, Carla I.Benzeroual, Kenza E.Merchant, AnwarBerens, Pamela D.Merkiel-Pawłowska, SylwiaBergdahl, AndreasMero, AnttiBerger, Mette M.Merritt, MatthewBergheim, InaMessa, Pier GiorgioBerglund, StaffanMessaritakis, IppokratisBergström, AnnaMessier, ClaudeBering, Stine BrandtMettler, SamuelBerkley, James A.Meyer, BarbaraBernard, JonathanMeyer, Katie A.Bernardo, Ana PaulaMeynier, AnneBernasconi, PiaMeyre, DavidBernhard, WolfgangMian, CaterinaBerni Canani, RobertoMicek, AgnieszkaBernlohr, David A.Michaelidou, Alexandra-MariaBernstein, MelissaMichalkiewicz, MarthaBerretta, MassimilianoMichels, Alexander J.Berrington, JanetMichigami, ToshimiBerry, MarlaMicol, VicenteBerry, NarelleMidgley, AdamBertolo, RobertMiele, LucaBertram, Hanne ChristineMielenz, ManfredBest, KarenMielgo Ayuso, JuanBeto, Judith A.Mietlicki-Baase, Elizabeth G.Bettaieb, AhmedMigliaccio, SilviaBeverly, Levi J.Mikhailov, Theresa A.Bezirtzoglou, EugeniaMiki, YasuhiroBharadwaj, ShishiraMilan, AmberBhatia, JatinderMilkovska-Stamenova, SanjaBhattacharjee, SonaliMillen, Amy E.Bhattacharya, SayakMiller, Dr. MichaelBhavsar, Amit P.Miller, JacquelineBiank, Vincent F.Miller, MarkBidel, SiamakMills, Kerry E.Bidwell, Amy J. Minami, AkiraBienertova-Vasku, JulieMinato, Ken-ichiroBiesiekierski, JessicaMinder, Elisabeth I.Bilal, UsamaMineo, ChiekoBingham, RichardMinicucci, Marcos F. Birch, LauraMinisola, SalvatoreBird, AmandaMinjares-Fuentes, Jose RafaelBirrell, MarkMioso, RobertoBisanz, JordanMiotto, PaulaBishayee, AnupanMiralles, BeatrizBishop, KarenMiranda, JonatanBittinger, KyleMiranda, Jose M.Bitto, AlessandraMirmiran, ParvinBix, LauraMisciagna, GiovanniBjørke Monsen, Anne-LiseMistretta, AntonioBjørndal, BodilMistura, LorenzaBlachier, FrançoisMiura, KyokoBlack, LucindaMiyata, YasuyoshiBlack, Maureen M.Miyazaki, TeruoBlais, AnneMiyazaki, TetsuroBlando, FedericaMiyazaki, ToruBlaner, William S.Miyoshi, EijiBlanton, CynthiaMizumura, KazueBlesso, ChristopherMizuno, KatsumiBlock, GaldysMizuno, MasashiBo, SimonaMladěnka, PřemyslBoard, MaryMlakar, VidBoaz, MonaMocanu, VeronicaBodet, CharlesMoestrup, Søren KraghBoekema, BoukeMohajeri, M. HasanBogl, Leonie HelenMohamed, SuhailaBøgwald, JarlMolin, MarianneBøhn, Siv K.Mollen, Kevin P.Boland, MichaelMond, JonBonaccio, MarialauraMonda, VincenzoBonaccorsi, GuglielmoMonti, Daria MariaBonavina, LuigiMontilla, AntoniaBondonno, Catherine P.Montoya, Carlos A.Bondonno, Nicola P.Moon, YuseokBondy, Stephen C.Moor, Molly A.Bongers, KaleMorabito, AntoninoBonilla Ocampo, Diego A.Morabito, RossanaBonilla, CarolinaMorales Suárez-Varela, María M.Bonomini, FrancescaMorán, José M.Bonos, EleftheriosMorán, LaraBoquien, Clair-yvesMoranta, DavidBora, Puran S.Moreau, RegisBorbély, YvesMorelli, LorenzoBorengasser, Sarah J.Moreno, Diego A.Borer, Katarina T.Moretti, DiegoBosch Ruiz, MarcMorganti, PierfrancescoBosch, ChristineMori, KiyoshiBosnjak, BerislavMori, NaokiBotelho, GoretiMorioka, TomoakiBotham, KathleenMorisset, Anne-SophieBottoms, LindsayMorris, Claudia R.Boucher, Barbara J.Morris, MelanieBoushey, CarolMorse, Nancy L.Bowen, Deborah J.Mosawy, SaphaBowling, AprilMoser, OthmarBoyer, ConstanceMosoni, LaurentBoyle, Robert J.Most, JasperBradbury, Kathryn E.Moughan, Paul J.Bradshaw, PatrickMougiakakou, StavroulaBrandhagen, MartinMoukarzel, SaraBrandt, KirstenMoulder, John E.Brantsæter, Anne LiseMoulis, Jean-MarcBranum, Amy M.Mounier, CatherineBraun, Kim V. E.Mountjoy, MargoBravenboer, NathalieMuhsen, KhitamBravo, LauraMukhopadhyay, ParthaBray, GeorgeMulhern, Maria S.Brecht, MarcusMulinacci, NadiaBreen, CathyMüller, Manfred JamesBreuillard, CharlotteMüller, ThomasBrewer, George J.Mulligan, Angela A.Brewster, Lizzy M.Mulvihill, ErinBriassoulis, GeorgeMunblit, DanielBriend, AndréMuñiz, PilarBriggs, Marc A.Muñoz-Alférez, M. JoséBrighenti, SusannaMurach, KevinBril, FernandoMurgia, ChiaraBrinkley, TinaMuriel, PabloBriskey, DavidMurotomi, KazutoshiBroderick, Tom L.Mursu, JaakkoBrookes, Denise S. K.Murthi, PadmaBroom, IainMuscaritoli, MaurizioBrosnihan, K. BridgetMusumeci, GiuseppeBroszczak, DanielMuszyńska, BożenaBrottveit, MargitMwinyi, JessicaBrough, LouiseMyneni, AjayBrown, KatieMyte, RobinBrown, Kyle E.Na, HuiminBrown, LaVerne L.Na, Hye-KyungBrown, LeanneNagai, KatshitoBrown, PaulaNagai, YoshinoriBrown, RachelNagao, KenjiBrownell, ElizabethNagaraj, ChandranBrownlee, Iain A.Nagasawa, HerbertBruce-Keller, Annadora J.Nagata, SatoruBruckbauer, AntjeNair-Shalliker, VisaliniBruneau, Michael Jr.Naito, YujiBruns, Danielle R. Nakahama, Ken-ichiBruyndonckx, LucNakajima, AtsushiBuccigrossi, VittoriaNakayama, MasaruBucher, TamaraNakhauka, Ekesa BeatriceBuckland, NicolaNamork, EllenBuddington, RandalNaninck, Eva F. G.Buechler, ChristaNanno, MasanobuBügel, SusanneNaruishi, KojiBugliani, MarcoNasser, JenniferBührer, ChristophNatarajan, Sathish KumarBukong, Terence N.Naumovski, NenadBunn, DianeNavarrete-Muñoz, Eva MariaBurden, RichardNavarro, EstanisBurgess, John R.Navarro, VirginiaBurghardt, Kyle JonNavarro-Alarcon, MiguelBurkhart, SarahNavas-Carretero, SantiagoBurkhead, JasonNaviglio, SilvioBurns, DavidNeacsu, MadalinaBurri, Betty J.Neale, ElizabethBurrows, TracyNeale, Rachel E.Burtey, StéphaneNeele, DellschaftBurton, E. ThomaseoNeelis, Esther G.Burton, Jeremy P.Neely, GregBuscail, CamilleNegro, MassimoButel, Marie-JoséNeilson, AndrewButler, TomNelson, GreggButterworth, Peter J.Nenna, RaffaellaBuyken, Anette E.Nestel, PaulByberg, LiisaNeto, António W. GomesBybrant, Mara CerqueiroNeupane, Sudan PrasadByrne, RebeccaNewby, RuthCabanillas, BeatrizNewnham, EvanCabezas, Manuel CastroNguyen, Tanya T.Cadario, FrancescoNi Mhurchu, ClionaCadeau, ClaireNicklas, TeresaCaffarelli, CarloNie, PengCai, DeminNielsen, ForrestCai, HuiNielsen, Ole HaagenCai, LuNieman, David C.Cai, ShirongNiemeier, AndreasCairrão, ElisaNieto, GemaCalani, LucaNieves, Jeri W.Calder, PhilipNikawa, TakeshiCaldovic, LjubicaNiles, Richard M.Calkins, Kara L.Nilsson, AkeCamire, Mary EllenNishi, Stephanie K.Campbell, Matthew DNissensohn, MarielaCamps, JordiNistala, RaviCanella, Daniela SilvaNobles, JamesCanfora, Emanuel E.Nogués, M. RosaCannell, John J.Nohr, DonatusCapaldo, BrunellaNolden, AlissaCapel, FrédéricNomikos, TzortzisCapewell, SimonNomura, YoshihiroCapitanio, NazzarenoNonn, LarissaCaporaso, NicolaNonnemacher, Michael R.Capuani, SilviaNordgren, TaraCardenas, AndresNorsa, LorenzoCardillo, CarmineNorval, MaryCardoso, LuísNoubissi, FeliciteCardoso, SusanaNovellino, EttoreCarlberg, CarstenNowak, Felicia V.Carlsen, Monica H.Nowicka, GrażynaCarnevale, DanielaNowson, CarylCarocho, MárcioNtalli, Nikoletta G.Caroli, Anna.Nualart, FranciscoCaron, Joan McIntyreNugent, AnneCarpene, ChristianNumazawa, SatoshiCarr, William HNybacka, SannaCarradori, SimoneO’Brien, Sarah H.Carrillo-Sepulveda, Maria AliciaO’Connor, EibhlísCasanovas, CarmenO’Gallagher, KevinCascone, SaraO’Hallora, Siobhan A.Casperson, Shanon L.O’kane, GabrielleCassetta, LucaO’Keefe, Stephen JDCastell, LindyO’Mahony, LiamCastellazzi, Anna MariaO’Malley, DervlaCastello, AdelaO’Tierney-Ginn, PerrieCastrogiovanni, PaolaOaks, Brietta M.Catalano, AntoninoObana, AkiraCatassi, CarloOdland, Jon ØyvindCauli, OmarOgawa, YukiharuCeccarelli, SaraOh, Sang-HoCeolotto, GiulioOhlemiller, Kevin K.César, CarinaOhlsson, BodilCesareo, RobertoOhnishi, MasatoshiChachay, Veronique S.Öhrvik, VeronicaChae, HeeyoungOhta, TakeshiChambers, Edgar IVOjala, Johanna O.Chan, Catherine B.Ojalvo, Daniel LozanoChan, JulieOjima, KoichiChan, MariaOka, ShuntaroChand, SourabhOkuda, MasayukiChandra-Hioe, Maria VeronicaOkuda, NagakoChang, Chen-KangOlde Rikkert, Marcel G. M.Chang, Chuang-RungOldewage-Theron, WilnaChang, Eugene B.Olendzki, BarbaraChang, Fang-RongOliveira, KarlianaChang, Hsin-IOliveira, Paula Alexandra Martins De Chang, Jer-MingOliveras-López, María-JesúsChang, Jung-SuOliviero, TeresaChang, Sue-JoanOlmedilla-Alonso, BegoñaChao, Ariana M.Olney, Deanna K.Chapple, SarahOlsen, SjurðurChassard, ChristopheOlza, JosuneChaung, Hso-ChiOmagari, KatsuhisaChauveau, PhilippeOmar, Syed HarisChavarro, JorgeOmotayo, MoshoodChaves-Lopez, ClemenciaOms-Oliu, GemmaChen, EuniceOnyenwoke, Rob U.Chen, GuibingOpperhuizen, AnneloesChen, GuoxunOrenes-Piñero, EstebanChen, Jih-JungOrmsbee, MichaelChen, Ling-WeiOrtega, Francisco JoséChen, QiOrtiz, AlbertoChen, Ruei-mingOsborne, BrennaChen, Shiu-NanOskarsson, ViktorChen, ShuaiÖstenson, Claes-göranChen, Szu-ChiaOta, TsuguhiroCheng, I-ShiungOtsuki, TakemiCheng, Juei-tangOu, Bor-rungCheng, KenOude Griep, Linda M.Cheng, Kuang-HungOuguerram, KhadijaCheng, Nai-ChenOvervad, Thure FilskovCHENG, Wen-HsingOzawa, MioCheng, XianwuPadrão, PatríciaChengwen, SunPagonopoulou, OlgaCherasse, YoanPaik, JisunCherbuy, ClairePainter, Jodie N.Cherniack, Evan PaulPal, AbhijeetCheungpasitporn, WisitPala, ValeriaChiang, Chien-HsiehPaller, ChanningChiang, Meng-TsanPalmer, Amanda C.Chiarioni, GiuseppePammi, MohanChiavaroli, LauraPan, MeixiaChiechio, SantinaPan, Min-HsiungChien, Yi-WenPanagiotidis, MihalisChieppa, MarcelloPanaro, Maria AntoniettaChilibeck, PhilPanza, FrancescoChiou, Wen-FeiPanzella, LuciaChirumbolo, SalvatorePaoli, AntonioChitchumroonchokchai, ChureepornPaolo, ArosioChiurchiù, ValerioPapadakis, SophiaChmurzyńska, AgataPapadimitriou, AnastasiosCho, ClarePapadimitriou, LamprosCho, JinmyoungPapadimitriuou, KonstantinosCho, Ssang-GooPapanikolaou, YanniChobot, VladimirPapetti, AdeleChoi, Myung-SookParakhonskiy, BogdanChong, Mary Foong-FongParhofer, Klaus G.Choudhury, MalayPark, JunsooChristen, William G.Park, Yong SeekChristides, TatianaPark, YongsoonChristmann, ViolaParkar, Shanthi G.Christodoulou, Maria-IoannaParker, HelenChristophersen, ClausParker, Leslie A.Chruszcz, MaksymilianParlesak, AlexandrChu, AnnaParnell, JillChu, ShidongParodi, EmiliaChua, Wei-HangParola, MaurizioChun, Ock K.Parolini, CinziaChun, ReneParr, EvelynChung, Louisa Ming YanPasco, David S.Ciacci, CarolinaPascual-Teresa, SoniaCibulskis, CatherinePasinetti, GiulioCiccocioppo, RachelePasqui, FrancescaCicero, ArrigoPassamonti, SabinaCiclitira, PaulPassarella, DanieleCifelli, Christopher J.Passaro, AngelinaCifuentes, LilianaPatel, Yashomati M.Cione, ErikaPatinen, PerttiClark, WilliamPatole, SanjayClarke, NeilPaton, ChadClayton, DavidPazdro, RobertClemens, RogerPearce, ElizabethClemens, ZsófiaPeart, Daniel J.Clemente, Maria GraziaPedraza-Chaverri, JoséClifton-Bligh, Phillip BPedrera-Zamorano, Juan D.Coates, PenelopePedret, AnnaCochran, BlakePeery, Anne F.Cohen, RonaldPeiris, MadushaCollado, Pilar SánchezPellegrino, DanielaColleran, Heather L.Pelly, FionaCollings, PaulPeluso, IlariaColussi, GianLucaPeña Quintana, LuisComan, Maria MagdalenaPeña, Amado SalvadorCombet, EmiliePeña, Maria Marjorette O.Comelli, ElenaPenagini, FrancescaConceição, EvaPena-Rosas, Juan PabloConcors, Seth JasonPenas, Sonia EirasConrad, ZachPence, BrandtConsidine, RobertPeng, Chiung–HueiConti, PioPeng, LifengCooke, MattPeng, Wen-HuangCoppedè, FabioPentieva, KristinaCordaro, MarikaPenugonda, KavithaCordero, PaulPepe, SalvatoreCorish, ClarePepino, Marta YaninaCorley, Michelle M.Peppelenbosch, MaikelCornelis, Marilyn C.Percival, SusanCorniola, RikkiPereira, DavidCornish, M. LynnPereira, DoraCornish, StephenPereira, LeonelCorpe, ChristopherPereira-da-Silva, LuísCortés-Castell, ErnestoPerez, AntonioCorthay, AlexandrePerez, JaraCostantini, SusanPerez-Cano, FranciscoCosta-Rodrigues, JoãoPerez-Cornago, AuroraCostello, Rebecca BortzPerez-Stable, CarlosCoughlan, Melinda T.Periago, María JesúsCourtney-Martin, GlendaPerry, TracyCovasa, MihaiPeters, John C.Cravero, Maria CarlaPeters, Rachel L.Crespo, IrenePeters, SimoneCroft, KevinPetersen, Kristina S.Crossland, NicolaPeterson, Catherine A.Cubero, Francisco JavierPeterson, JuliaCullers, AndreaPetroff, Brian K.Cullerton, KatherinePettersen, CarolineCumming, Kristoffer T.Pettinato, GiuseppeCunha, RodrigoPeyer, KarissaCunningham-Rundles, SusannaPezzotto, Stella M.Cutuli, DeboraPhang, MelindaCzikora, IstvanPhilips, NeenaD’Addario, ClaudioPhillips, Mary E.D’Alessio, Patrizia A.Piccoli, Giorgina BarbaraD’archivio, MassimoPiccolo, BrianD’Auria, EnzaPiche, Marie-EveD’Auria, Maria ValeriaPicklo, Matthew J.Da Paz, Vanessa Ribeiro FigliuoloPiemontese, LucaDahech, ImenPierce, GrantDalbeni, AndreaPietrella, DonatellaDalbo, VincentPietrobelli, AngeloDall’Acqua, StefanoPignitter, MarcDalla Rosa, MarcoPignotti, GiselleDalmas, ElisePiknova, BarboraDalsgaard, Trine KastrupPilon, MarcDalton, CarolinePilz, StefanDanesi, FrancescaPinent, MontserratDankel, Simon N.Pirola, LucianoDarling, Andrea L.Piso, Rein JanDarmon, NicolePiva, TerrenceDarvin, MaximPivovarova, OlgaDas, AmitavaPlaza-Díaz, JulioDas, UndurtiPlazas Ávila, Maria De La ODashti, HassanPlochocki, Jeffrey H.Davidson, RosePlösch, TorstenDavila, Anne-MariePolanska, KingaDavinelli, SergioPolidori, Maria CristinaDavison, KarenPollock, Richard L.Davy, BrendaPoole, David C.Davy, Kevin P.Popovich, DavidDawood, Mahmoud A. O.Poquet, LaureDe Borst, Martin H.Porres, Jesús M.De Cock, NathaliePortengen, LützenDe Geest, BartPorter, Judi A.De Gruijl, FrankPosovszky, CarstenDe Kreutzenberg, SaulaPotenza, LuciaDe La Cruz, José PedroPoti, JenniferDe La Monte, SuzannePotischman, NancyDe La Parra, ColumbaPotter, JuliaDe Las Heras, JavierPotthoff, Matthew J.De Leo, VincenzoPoudyal, HemantDe Lorgeril, MichelPourshahidi, KirstyDe Magistris, LauraPower, MadeleineDe Magistris, TizianaPowers, Jacquelyn M.De Mestral Vargas, CarlosPowers, James S.De Pascual-Teresa, SoniaPowles, JohnDe Smet, StefaanPozzilli, PaoloDe Sotomayor, Maria AlvarezPravst, IgorDe Steur, HansPreitner, FredericDe Vito, ElisabettaPremkumar, MuralidharDe Vlieger, Nienke Pribus, PeterDe Vries, JeannePrieto-Lloret, JesusDe Wild, Victoire W. T.Primeaux, Stefany D.De Zordi, NicolaProbert, ChrisDean, LisaProestos, CharalamposDe-Bandt, Jean-PascalPugliese, GiuseppeDeBusk, RuthPurchas, Roger W.Deeth, Hilton C.Puri, Basant K.Dekker, MarloesPursey, KirrillyDel Bo, CristianPurwaha, PreetiDel Giudice, RitaPüschel, Gerhard P.Del Rio, DanieleQasem, Wafaa A.DeLany, JamesQin, BoDelarue, JacquesQuadros, Edward V.Deli, Chariklia K.Quigley, EamonDelisle, HélèneQuintero-Florez, AngelicaDella Torre, SaraRaatz, Susan K.Dellavalle, Diane M.Rachaputi, Rao C. N.Delmastro-Greenwood, MeghanRachek, LyudmilaDelvin, Edgard E.Rachoń, DominikDen Hartigh, Laura J.Radmacher, PaulaDenham, Bryan E.Radyukina, N. L.Deo, PermalRaimann, Jochen G.Depner, Christopher MichaelRainger, George EdwardDepoortere, IngeRajakumar, KumaravelDerave, WimRaju, RaghavanDerraik, José G. B.Rallabhandi, PrasadDesai, Mahesh S.Raman, JayDeschasaux, MélanieRamdath, DanDesprés, Jean-PierreRamos Campo, Domingo JesúsDesselberger, UlrichRamos, SoniaDetzel, PatrickRana, Brinda K.Deutsch, JonathanRanganathan, NatarajanDeutz, Nicolaas E. P.Ranjit, NaliniDev, DiptiRanzato, EliaDevesa, VicentaRao, ShripadaDevlin, AngelaRapoport, Stanley I.Dey, MoulRathinavelu, AppuDi Biase, StefanoRattan, SureshDi Daniele, NicolaRavallec, RozennDi Felice, GabriellaRay, SeemunDi Gioia, DianaRaz, OlgaDi Iorio, Biagio RaffaeleReaney, Martin J. T.Di Nardo, GiovanniRees, BillDi Palma, MichaelReeve, BelindaDickerson, Roland N.Reeve, VivienneDickinson, KacieRege, ShraddhaDilger, RyanReger, MichaelDillon, E. LicharRehm, ColinDinesen, Pia ThistedReicks, MarlaDinh, Dzung H.Reidlinger, DianneDiNicolantonio, James J.Reimer, RayleneDinu, MonicaReinblatt, Shauna P.Dioum, Elhadji M.Reiner, MartinDjuric, ZoraReis, FlávioDobrosielski, Devon A.Reiser, JochenDoig, Gordon S.Reisman, Scott A.Dolinsky, Vernon W.Reitelseder, SørenDominguez, Ligia J.Reiter, E. MirandaDominguez-Perles, RaulRemacha, Angel F.Donadio, CarloRen, JianguoDonne, BernardRenaud, JustineDonno, DarioRenner, WilfriedDonofry, Shannon D.Repa, AndreasDonovan, SharonResch, BernhardDoshi, SimitResiere, DaborDossus, LaureRevuelta Iniesta, RaquelDötsch-Klerk, MariskaRey, FedericoDoyle, ToddRezamand, PedramDragsted, Lars OveRezzani, RitaDreher, MarkRhodes, JonathanDrevon, Christian ARibas-Barba, LourdesDrew, Janice E.Ricciardelli, CarmelaDrobnic, FranchekRichard, CarolineDrossard, ClaudiaRichardson, AlanDuckworth, Lauren C.Riddlle, LynnDufour, Darna L.Riedel, BernhardDullaart, Robin P. F.Riedel, Christian U.Dumler, FrancisRienecke, ReneeDumont, PatrickRiesco, EléonorDunford, ElizabethRigo, JacquesDuntas, LeonidasRigottier-Gois, LionelDurazo-Arvizú, Ramón AngelRiis, SimonDurazzo, AlessandraRiley, MalcolmDuring, AlexandrineRimbach, GeraldDuryee, Michael J.Ringel-Kulka, TamarDusso, AdrianaRink, LotharDuwaerts, CarolineRios-Covian, DavidEarnest, Conrad PRiscuta, GabrielaEastman, CreswellRivellese, Angela A.Eaton, PhilipRivero-Pérez, M. DoloresEbeling, PeterRizzarelli, EnricoEduardo-Figueira, MariaRizzo, Angela MariaEdurne, Simón MagroRobberecht, HarryEfird, JimmyRobert-Da Silva, ChristineEgger, GarryRoberts, Rosebud O.Ehret, Georg B.Robertson, DeniseEinöther, Suzanne J. L.Robertson, JeremyEkuni, DaisukeRobertson, KirstenElaine Hardman, W.Robitaille, JulieEldridge, Alison L.Robles, BrendaEleftheriadis, TheodorosRobson, Shannon M.Elisabet, ForsumRodrigues, IsabelEllervik, ChristinaRodriguez Artalejo, FernandoElli, LucaRodríguez, Alexander J.Elli, MarinaRodríguez, María CorreaElliott, Michael B.Rodríguez, MarianoEl-Salhy, MagdyRodriguez-Herrera, AlfonsoEl-Seedi, Hesham R.Rodriguez-Mateos, AnaElsheikha, Hany M.Roe, Brianel-Sohemy, AhmedRoelofs, EricaEmpl, MichaelRoggero, PaolaEngle-Stone, ReinaRohner, FabianEnnis, Julie MasonRohrmann, SabineEnomoto, HirayukiRomanelli, FrancescoErdman, JohnRomani, AndreaEri, RajRomeo, StefanoEri, RajaramanRonco, PierreEricsson, MadeleneRondanelli, MariangelaEriksson, Johan G.Root, Martin M.Ershow, Abby G.Rosado, CatarinaErzinger, MelanieRose, DevinEscolà-Gil, Joan CarlesRosendahl, JonasEsposito, CiroRosendale, Douglas IanEsworthy, SteveRosenlund, HelenEuston, StephenRosenthal, PhilipEvans, Christian C.Rosinger, AsherEvans, E WhitneyRoss, AlastairEvans, GethinRosset, RobinEvans, Joseph L.Rossi, FilippoEveraert, NadiaRossi, MauroEvin, GenevièveRossi, PaolaEyles, Darryl W.Rossiter, JohnEzedine, BouhlelRostaing, LionelEzekiel, UthayashankerRostami, KamranFabbricatore, MariantoniettaRother, KristinaFabiani, RobertoRouhiainen, AriFactor-Litvak, PamRousseau, GuyFagerstedt, KurtRousselot, BonnefontFahey, GeorgeRowland, IanFairchild, Timothy JohnRoychowdhury, SanjoyFairweather, StephenRoza, Sabine J.Fairweather-Tait, Susan J.Rozen, RimaFang, JimRozhon, WilfriedFanizzi, Francesco P.Rubio-Arraez, SusanaFargion, SilviaRucklidge, JuliaFarhat, GraceRuddock, HelenFarina, Emily K.Rudkowska, IwonaFarr, Susan A.Rudolph, BryanFarran-Codina, AndreuRudolph, Michael C.Farrar, MarkRudzitis-Auth, JeannetteFayet-Moore, FlaviaRufian Henares, Josè AngelFeas, XesusRuscica, MassimilianoFedosov, SergeyRush, Elaine CFeinman, Richard D.Russell, Alexandra C.Felder, Robin A.Russell, Fraser D.Feldman, DavidRussell, MarkFelt, BarbaraRutegård, MartinFeng, YibinSaarela, MariaFenton, TanisSabate, JuanFeo, FrancescoSacks, GordonFeresin, Rafaela G.Sadanaga, TsuneakiFerlazzo, NadiaSaha, ShyamaliFernandes, EduardaSahay, BikashFernandes, Maria FernandaSaikumar, PothanaFernandez, Maria LuzSainsbury, KirbyFernandez-Aranda, FernandoSaisho, YoshifumiFernández-Sánchez, María LuisaSakhaee, KhashayarFerramosca, AlessandraSala, RobertoFerrario, ChiaraSalaheen, SerajusFerraris, Ronaldo P.Salehi, AhmadFerraro, Pietro ManuelSalim, SaminaFerreira, IsabelSalis, AmandaFerreira, Rui M.Salles, ChristianFerrell, Jessica M.Salmerón, IvanFerrer-García, MartaSalomone, FedericoFerrie, SuzieSalvatore, FrancescoFetissov, Sergueï O.Salvo, AndreaFetterman, James W.Samala, Niharika R.Fiaccadori, EnricoSamaniego Vaesken, María De LourdesField, CatherineSamuel, ChrishanFields, DavidSamuelsson, Anne-MajFildes, AlisonSan Martin, CarmenFiller, GuidoSanchez, ChristelleFine, Eugene J.Sánchez-Fidalgo, SusanaFine, James BurkeSanchez-Muniz, Francisco J.Fineschi, VittorioSandell, MariFinley, JohnSanders, Kerrie M.Fiocchi, AlessandroSanders, TomFiocco, DanielaSandvik, PernillaFirrman, Jenni AnnSankavaram, KavithaFlack, Kyle D.Sanmartin, ChiaraFlechtner-Mors, MarionSanté-Lhoutelliera, VeroniqueFleenor, Bradley S.Santoro, LucaFleig, LenaSantoro, NicolaFlexeder, ClaudiaSantos, DeboraFloch, Martin H.Santulli, GaetanoFlueck, Joelle L.Sanz-Paris, AlejandroFlynn, Charles R.Sarris, JeromeFoegeding, E. AllenSassi, YassineFogelholm, MikaelSastre-Serra, JorgeFölster-Holst, ReginaSato, DaisukeFontana, LuisSato, KenjiFoong, Rachel E.Sato, ShinFoote, Janet A.Saturnino, CarmelaForbes, Scott. C.Sauder, Katherine AnnForman, MicheleSaunders, BryanForrest, LaurenSavary-Auzeloux, IsabelleForsmark, Chris E.Savion, NaphtaliForsyth, AdrienneSaw, Constance L. L.Fortenberry, MeganSayón-Orea, CarmenFoster, Charles B.Scano, PaolaFoster, MeikaScaramuzza, Andrea E.Fouladkhah, AliyarScarmeas, NikolaosFourtounas, CostasScazzina, FrancescaFox, SimonSchachter, MichaelFragkos, KonstantinosSchag, KathrinFragopoulou, ElizabethSchauss, AlexanderFrame, Leigh A.Scheers, Nathalie M.Franco, CarlosScheffler, ChristianeFranklin, PeterScherf, KatharinaFranko, AndrasSchiavone, StefaniaFrazer, DavidSchild, LorenzFrazzi, RaffaeleSchini-Kerth, ValérieFreeman, Linnea R.Schirmer, BastianFreije, José M. P.Schlegel, AmnonFriel, JamesSchloss, Janet M.Friesen, CarolSchmetterer, LeopoldFu, Tzu-FunSchmidt, RicardaFuge, RonSchnackenberg, LauraFujihara, HisakoSchoeler, Natasha E.Fujimori, KoSchoeller, DaleFujita, MikakoSchoenaker, Danielle A. J. M.Fukata, MasayukiSchonthal, AxelFukuda, MitsuruSchulz, Kalynn M.Fukuda, YoshiharuSchulze, Kerry J.Fukui, MichiakiSchupp, MichaelFulgoni, Victor L. IIISchwab, UrsulaFullerton, MorganSchwartz, Gary G.Funato, HiromasaSchwetz, VerenaFung, Ellen B.Scicchitano, PietroFürnsinn, ClemensScoffield, JessicaFusaro, MariaScott, Fraser W.Gachon, FredericScott, SamGadgil, Meghana D.Scovassi, Anna IvanaGahche, JaimeScragg, RobertGalanis, AlexScudiero, OlgaGaleazzi, Gian MariaSculley, Dean V.Galli, FrancescoSeal, JudyGalvano, FabioSeale, LuciaGálvez, JulioSearle, Samuel D.Gan, Ren-YouSears, BarryGanapathy, VadivelSebastian, RhondaGander, JenniferSechi, Leonardo A.Gandy, JoanSeddon, Johanna M.Gangloff, AnneSegovia-Siapco, GinaGanji, VijaySekikawa, AkiraGao, JieSekulic, DamirGarcia De La Hera, ManoliSellayah, DyanGarcia, AdaSelmin, Ornella I.García, Marta MesíasSelvam, ChelliahGarcia-Bailo, BibianaSena, Cristina M.García-Martínez, EvaSenchina, DavidGarcía-Parrilla, Maria.C.Serio, FrancescoGarcía-Sánchez, AsunciónSerra, AndreaGard, PaulSerra, DolorsGarden, FrancesSerra, FranciscaGarden-Robinson, JulieSerralheiro, Maria L.Garg, ManoharSerra-Majem, LluisGarnacho-Castaño, Manuel VicenteSerrano, Jose C. E.Garza, KimberlySerrat, Maria A.Gasparrini, MassimilianoSethi, GautamGassler, NikolausSetzer, William N.Gaudette, Nicole J.Seubert, John M.Gaudichon, ClaireSeyssel, KevinGaultier, AlbanSha, ZheGay-Quéheillard, JeromeShackelford, Rodney E.Ge, DongxiaShah, DilipGe, XiaodongShah, RominaGea, AlfredoShah, Savit. H.Gebhardt, RolfShah, ZahoorGeisler, CorinnaShahar, DanitGenoni, AngelaShaikh, Saame RazaGentili, SheridanShakibaei, MehdiGeorgiev, VasilShanely, R. AndrewGérard, PhilippeShankar, EswarGerards, SanneShankar, PadminiGerber, Leonard E.Shao, AndrewGerber, MarietteShapses, SueGhassabian, AkhgarSharma, BheshGhatak, ArindamSharma, MukutGiaginis, ConstantinosSharma, NaveenGiamarellos-Bourboulis, Evangelos J. Sharma, RohitGiampieri, FrancescaSharma, SushilGianfrani, CarmenSharma, UmakantGianini, Loren M.Sharp, PaulGiannenas, IliasShecterle, Linda M.Giannì, Maria LorellaShen, LeslieGiapros, VasileiosShen, QiangGiavaresi, GianlucaShen, Szu-ChuanGibney, EileenSheridan, Paul O.Gibson, Alice. A.Shertzer, Howard G.Gibson, PeterShewale, SwapnilGibson, SimoneShi, SuanGiel, KatrinShiao, Young-JiGiera, MartinShida, KanGifford, Janelle A.Shidoji, YoshihiroGiles, Grace E.Shimada, YasuhitoGill, TiffanyShimizu, MitsuruGillevet, PatrickShimizu, TakahikoGiordano, MauroShimosawa, TatsuoGiudetti, AnnaShin, Andrew C.Gkimpas, GeorgiosShin, DayeonGkolfakis, ParaskevasShipman, KateGłąbska, DominikaShirin, ZiaeiGlade, Michael J.Sholkamy, EmanGladine, CecileShukla, Alpana P.Glahn, RaySiega-Riz, Anna MariaGlaister, MarkSilswal, NeerupmaGlenn, Melissa J.Silva, Branca M.Glenn, Thomas C.Silva, RobinGo, Gwang-WoongSilverberg, Donald S.Godfrey, Henry P.Silvestre, Marta Filipa PaulinoGodos, JustynaSim, KyraGoetz, MargaretheSimko, FedorGoglia, FernandoSimon, Marie-ChristineGoicoa, TomásSimoni, JanGoldbohm, R. AlexandraSimopoulos, Artemis P.Gold-Smith, FuchsiaSimpson, Melanie RaeGoletzke, JaninaSinger, PierreGolley, RebeccaSingh, AnilGolovko, MikhailSingh, Ashok K.Gomez, NidiaSingh, AshutoushGómez-Ambrosi, JavierSingh, Chandra K.Gómez-Zorita, SaioaSingh, NagendraGomollon, FernandoSingh, Udai P.Gonçalves, GuilhermeSingh, Vijay P.Gonzalez, Adam M.Singh, VishalGonzalez, Javier T.Singhal, VibhaGonzález, SoniaSiniscalco, DarioGonzalez-Barcala, Francisco JavierSirtori, CesareGonzalez-Casanova, InesSjöberg, KlasGonzález-Reimers, EmilioSjostrom, Elisabeth StoltzGoodman, RichardSkaaby, TeaGorham, EdwardSkeaff, SheilaGorman, ShelleySkelton, Matthew R.Gower, BarbaraSlater, JoyceGoya, LuisSleigh, AlisonGrace-Farfaglia, PatriciaSłoczyńska, KarolinaGraff, Rebecca E.Sluik, DiewertjeGraham, Daniel J.Sluimer, JudithGramazio, PietroSmith, CarenGramlich, LeahSmith, ClaireGranados-Principal, SergioSmith, GordonGrant, WilliamSmith, Jill P.Grasa, LauraSmith, MichaelGrases, FelixSmith, TarynGrasselli, ElenaSmoliga, JamesGrech, AmandaSmutzer, GregoryGreen, PeterSnaedal, SunnaGreene, Michael W.Snih, Soham AlGreenhaff, PaulSoares, MarioGreenhalgh, DavidSocha, KatarzynaGreenway, Frank L.Söderström, LisaGreenwood, Darren C.Soenen, StijnGreenwood, Michael T.Sohn, Uy DongGregori, DarioSokół-Łętowska, AnnaGregory, JesseSolà Alberich, RosaGreibe, EvaSolah, Vicky A.Gresham, EllieSolanas, MontserratGrether-Beck, SusanneSomoza, BeatrizGrider, ArthurSong, JaewhanGrieger, Jessica A.Song, Moon K. Griffin, IanSong, Sang-WookGriffith, MaySong, YoonJuGrilli, EsterSonoyama, KeiGrim, Clarence E.Sopade, Peter A.Grimes, CarleySoraisham, Amuchou S.Grisk, OlafSoreq, HermonaGrootaert, CharlotteSornay-Rendu, ElisabethGrosso, ClaraSotomayor, Camilo G.Grosso, GiuseppeSotos-Prieto, MercedesGrundy, Myriam M. L.Soueidan, AssemGrungreiff, KurtSoulage, ChristopheGrünler, JacobSourij, HaraldGu, FengSouza-Smith, FlaviaGuarino, AlfredoSoybel, David I.Guarino, MariaSpegazzini, NicolasGuasch-Ferre, MartaSpégel, PeterGudbrandsen, Oddrun A.Spence, J. DavidGudey, Shyam KumarSpiro, AyelaGueant, Jean-LouisSpitznagel, Mary BethGueimonde, MiguelSpriet, LawrenceGuenther, Patricia M.Stacchiotti, AlessandraGuerre, PhilippeStadlbauer, VanessaGuglielmetti, SimoneStadler, Diane D.Gugliucci, AlejandroStafford, Lorenzo D.Guidetti, MargheritaStagos, DimitriosGuido, Luis F.Stamm, RosemaryGuiné, Raquel P. F.Stanforth, PhilGunst, JanStangl, GabrieleGuo, WeiminStanley, DraganaGupta, Priya M.Stanley, RogerGutiérrez-Juárez, RogerStannard, StephenGutierrez-Merino, CarlosStarek, MałgorzataGutiérrez-Orozco, FabiolaStarup-Linde, J.Haapala, Eero A.Stauber, Rudolf E.Haarmann-Stemmann, ThomasStaudacher, HeidiHaase, HajoStautz, KaidyHaboubi, NadimStea, Tonje H.Haczeyni, FahrettinSteenson, SimonHadjikakou, MichalisStefanidou, Maria E.Hadjisavvas, AndreasStefanon, BrunoHagenlocher, YvonneStefler, DenesHagman, EmiliaStegmayr, BerndHajduch, EricStehle, PeterHaldar, SumantoSteil, Garry M.Hall, Marcia.Stengel, AndreasHall, Ulrika AnderssonStephen, IanHalloran, Bernard P.Stevens, Paul E.Hamaguchi, MasahideStevens, RebekahHambidge, MichaelStewart, MariaHamden, KhaledSteyn, FrederikHamdy, OsamaStilli, DonatellaHamlin, RobertStobdan, TseringHamner, HeatherStokes, Caroline S.Hampel, DanielaStomby, AndreasHan, JaehongStork, Christian J.Han, Seung JinStough, CathleenHan, Sung NimStove, Christophe P.Han, ZhiyongStrasser, BarbaraHand, TimothyStratakis, NikosHaneklaus, MoritzStrazzullo, PasqualeHannibal, LucianaStrekalova, TatyanaHanson, CorrineStringham, James M.Hanson, Karla L.Stuart, SamHao, LeiStubbs, BriannaHara, HiroshiStuetz, WolfgangHardman, Roy J.Stull, AprilHardy, MelindaStumbo, Phyllis J.Haring, BernhardSu, Shu-JemHarnack, LisaSu, Xiao Q.Haron, MonaSubar, Amy F.Harris, MargaretSubhan, Fatheema BegumHarris, WilliamSui, ZhixianHarrison, FionaSukocheva, OlgaHart, Andrew R.Sukumar, DeepthaHart, KathrynSukumaran, Sunil KumarHart, Prue H.Suleria, Hafiz AnsarHärtel, ChristophSuliburska, JoannaHartmann, ChristinaSullivan, MargaretHartmann, PhillippSun, Feng-HuaHaschke, FerdinandSun, QianHasegawa, YukihiroSung, ValerieHashem, Kawther M.Suwa, MichihiroHashimoto, NaotoSuzuki, KatsuhikoHaskell-Ramsay, CrystalSweazea, KarenHassan, ImanŚwieca, MichałHassan-Smith, ZakiSylvetsky, AllisonHassouna, RimSzafron, MichaelHatsu, IreneSzászi, KatalinHatta, HideoSzepfalusi, ZsoltHawinkels, LukasSzewczyk, KatarzynaHay, PhillipaSzilagyi, AndrewHayes, AlanSzkudelski, TomaszHayes, Kenneth C.Szlagatys-Sidorkiewicz, AgnieszkaHaytowitz, David B.Szychlinska, Marta AnnaHearst, Mary O.Taghibiglou, ChangizHeathers, James A. J.Tahrani, Abd A.Hebbard, LionelTain, You-LinHeberden, ChristineTajiri, YujiHecht, JaquelineTakahama, UmeoHector, Amy J.Takahashi, SatoshiHedrick, ValisaTakahisa, KanekiyoHefferon, Kathleen LauraTakatani-Nakase, TomokaHeianza, YorikoTakaya, JunjiHeinen, MirjamTakayama, FusakoHeinonen, IlkkaTakechi, RyusukeHeiss, ChristianTakeda, EijiHekmatdoost, AzitaTakimoto, HidemiHeller, Martin C.Tako, EladHellmann, Jason L.Talati, ZenobiaHellwig, MichaelTamura, YukinoriHelms, Eric R.Tang, MinghuaHemilä, HarriTao-Hsin, TungHendrich, SuzanneTaoka, YousukeHendrie, GillyTappia, Paramjit S.Heneberg, PetrTappy, LucHennessy, ÁineTarantino, GiovanniHerman, MarkTarocchi, MirkoHernandez, MariaTasevska, NatashaHernandez, TeriTashiro, HirotakaHernández-Ledesma, BlancaTauler, PedroHerrera, EmilioTaupin, DougHerrick, KirstenTavernarakis, NektariosHess, SonjaTawia, SusanHewlings, Susan J.Taylor, Christopher A. Hida, AzumiTaylor, KathrynHidaka, Brandon H.Taylor, RachaelHider, RobertTaylor, T. MatthewHietikko, ElinaTe Morenga, LisaHigashida, KazuhikoTeas, JaneHiggins, MattTeasdale, ScottHildebrandt, HerbertTecce, Mario FeliceHiles, SarahTekle, MichaelHill Gallant, KathleenTelang, SuchetaHill, JamesTemeyer, Kevin B.Himotoe, TakashiTemme, Elisabeth H. M.Hioki, HirofumiTemple, NormanHirko, KellyTeodorowicz, MałgorzataHo, Chi-TangTepper, Beverly J.Ho, Su-ChenTer Horst, KasperHocher, BertholdTersey, Sarah A.Hodge, AllisonTeske, JenniferHodgson, JonathanTesoriere, GiovanniHoerr, RobertTessier, Frederic J.Hoerster, Katherine D.Teuber, RamonaHoffer, Leonard JohnTey, Siew LingHoffman, RichardThangaratinam, ShakilaHokama, TomikoThen, CorneliaHolick, MichaelThèvenod, FrankHolliday, AdrianThijs, CarelHolliday, Elizabeth G.Thomas, DianaHollis, BruceThomas, Helen E.Holmes, GeoffreyThomas, Lynn K.Holst, Jens JuulThompson, HenryHolst, MetteThomsen, HaukeHolt, Roberta R.Thomson, JasmineHolt, Tim A.Thornley, SimonHolvik, KristinThornley-Brown, DenyseHong, Mee YoungThunders, MichelleHönow, RuthThurnham, DavidHoogeveen, Ellen K.Thyagarajan, BaskaranHook, IngridTielemans, Myrte J.Hopcraft, Matthew S.Tierney, AudreyHorax, RonnyTimmerman, Kyle L.Horrigan, Louise A.Tinker, Sarah C.Hosick, PeterTintle, NathanHosohata, KeikoTipoe, GeorgeHossein-Nezhad, ArashTipton, KevinHotz, ChristineTissing, WimHou, Yu-ChihTitorenko, Vladimir I.Houston, Mark C.Titze, Jens MarcHowarth, GordonTiwari, SanjayHowe, AnnaToldo, StefanoHruz, Paul W.Toledo, AlvaroHsiang, Chien-YunToledo, EstefaniaHsiao, GeorgeToledo-Corral, ClaudiaHsieh, Shu-ChenTomasello, GiovanniHsieh, Tusty-JiuanTomaszewska, EwaHsu, Shu-haoTong, IrisHu, TianToorie, AnikaHuab, MarkTorquati, LucianaHuang, Chi-ChangTorres López, M^a^ IsabelHuang, Shih-YiTorres Perales, CarolinaHuang, ZhiTorres, SusanHuc, LaurenceToshimitsu, TakayukiHuggins, Catherine E.Tosi, PaolaHughes, CatherineToubekis, Argyris G.Hughes, DavidTouil-Boukoffa, ChafiaHughson, Michael D.Tournier, CaroleHull, Mark A.Tovar, JuscelinoHulthén, LenaTovoli, FrancescoHummel, SandraTownend, JonathanHuq, LailaTraber, MaretHur, Sun-JinTraina, GiovannaHuss, FredrikTran, CuongHuston, Robert K.Tran, PamelaHutchinson, JayneTranchant, CaroleHuysentruyt, KoenTraustadόttir, TinnaHypponen, ElinaTrehan, IndiIde, KazukiTremblay, AngeloIderaabdullah, Folami Y.Trenell, MichaelIdrus, NireliaTresserra-Rimbau, AnnaIizuka, KatsumiTrevena, HelenIjiri, DaichiTriaca, VivianaIJzerman, RichardTriantafyllou, KonstantinosIkegaya, NaokiTrico, DomenicoIlich-Ernst, JasminkaTrieu, KathyImai, ToshioTrif, MonicaImperatori, ClaudioTriunfo, StefaniaInagi, ReikoTroesch, BarbaraInestrosa, Nibaldo C.Troise, Antonio DarioInman, Denise M.Trojanowicz, BoguszIossa, SusannaTrombetta, DomenicoIqbal, Tariq H.Trommelen, JornIrini, AthanasakiTronel, ClaireIrwin, ChrisTrovato, GuglielmoIshihara, HisamitsuTrovik, JoneIshihara, KengoTroy, LisaIshii, ShinyaTruong, KhoaIuliano, SandraTsai, JamesIves, StephenTsao, FrancisIwahori, ToshiyukiTschon, MatildeIwai, AtsushiTselepis, ChrisIwamori, MasaoTseng, Ching-JiunnIzawa, Kazuhiro P.Tseng, Tung-SungIzawa, TakeshiTsiani, EvangeliaJaakkola, JohannaTsivgoulis, GeorgiosJaaro-Peled, HannaTsuzuki, TsuyoshiJablonski, HeidrunTuck, CarolineJackson, William F.Tucker, Larry A.Jacobs, DavidTucker, RobinJacobs, RenéTueros, ItziarJadeja, RavirajsinhTufarelli, VincenzoJager-Wittenaar, HarrietTur, Josep AntoniJahan-Mihan, AlirezaTuresky, RobertJain, AjayTuriel, MaurizioJakobsen, JetteTurner, JustineJakubowicz, DanielaTurner, RaymondJalili, ThunderTurner, Russell T.James, Rob S.Turpin-Nolan, SarahJamieson, Jennifer A.Tursi, AntonioJanda, ElzbietaTuuri, GeorgiannaJandacek, Ronald J.Tuvdendorj, DemidmaaJang, Byoung KukTylavsky, Frances A.Jang, Hyeung-JinTyler, ChurchwardJanhonen, KristiinaTyson, Crystal CenellJape, GayatriTyson, Darren R.Jarvinen-Seppo, KirsiTyunin, Alexey P.Jean-Marc Tadié, Jean-MarcTzakos, Andreas G.Jeckel, Kimberly M.Tzanakis, Ioannis P.Jefferds, Maria ElenaTziomalos, KonstantinosJENA, PRASANTUbago-Guisado, EstherJenkins, DavidUberti, DanielaJensen, Megan E.Uccella, Nicola A.Jerebtsova, MarinaUchida, YoshikazuJerković, IgorUebanso, TakashiJessri, MahsaUehara, MarikoJeżewska-Zychowicz, MarzenaUehara, TomoyaJiao, LiUhal, Bruce D.Jin, RanUjcic-Voortman, JoanneJirillo, EmilioUlatowski, LynnJohn, Katherine A.Ullrich, ReinerJohnson, ClaireUmano, Giuseppina RosariaJohnson, ElizabethUnno, KeikoJohnson, IanUpadhyaya, JasbirJohnson, StuartUrrechaga, EloísaJohnston, CarolUusi-Rasi, KirstiJones, JulieUusitupa, MattiJones, KerryVajro, PietroJones, Peter J. H.Valacchi, GiuseppeJoo, Nam-seokValentão, PatríciaJooken, EtienneValentini, LuziaJoosten, Koen F.Valenzuela, RodrigoJordan, Jennifervan Baak, MarleenJose, Pedro A.Van Ballegooijen, Adriana J.Joven, JorgeVan Ballegooijen, HanneJówko, EwaVan Den Engel-Hoek, LenieJoy, Jordan M.Van Den Heuvel, Ellen GHMJuan, WenyenVan Der Haar, FritsJudd, SuzanneVan Der Kamp, Jan WillemJuge, NathalieVan Etten, JamieJukarainen, SakariVan Griensven, LeoJulia, ChantalVan Guelpen, BethanyJulve, JosepVan Raay, TerryJuonala, MarkusVan Schothorst, Evert M.Jurewitsch, BrianVan Strien, TatjanaJurikova, TundeVan Wijk, NickJuskiewicz, JerzyVan Zanten, Arthur R. H.Juszczak, LesławVance, David E.Jyväkorpi, Satu K.Vance, TerrenceKabil, OmerVandenboom, ReneKagiya, TadayoshiVandenplas, YvanKahal, HassanVander Wal, JillonKahleova, HanaVanderhoof, Jon A.Kaido, ToshimiVangelista, LucaKalanetra, KarenVarady, KristaKalhan, SatishVarela-López, AlfonsoKällén, BengtVarela-Moreiras, GregorioKalliopi, KotsaVargas, Jorge H.Kalogirou, CharisVarraso, RaphaëlleKaludercic, NinaVashist, Yogesh KumarKamada, NobuhikoVásquez, ElizabethKamat, PradipVassalle, CristinaKaminskyy, Vitaliy O.Vassallo, MatteoKamolz, LarsVaughan, Roger A.Kanai, YoshikatsuVaughn, MathewKancherla, VijayaVecchione, CarmineKandar, RomanVeerman, J. LennertKang, Jae SeungVeleeparambil, ManojKantarci, AlpdoganVenancio, Vinicius PaulaKao, Erl-ShyhVenema, KoenKapsokefalou, MariaVenkatadri, RajkumarKarachaliou, MariannaVenkatesha, ShivaprasadKarakochuk, CrystalVenn, BernardKaramichos, DimitriosVenter, CarinaKaratzi, KalliopiVentrella, DomenicoKarki, RajendraVenturi, FrancescaKarlsson, ErikVerain, Muriel C. D.Karras, SpirosVerbeke, KristenKasarda, DonaldVerd, SergioKašparovský, TomášVergara, DanieleKass, Lindsy S.Verkaik-Kloosterman, JannekeKatayama, ShigeruVetter, Stefan W.Kato, HiroyukiVetvicka, VaclavKato, HisanoriVia, Michael A.Kato, NorihisaVicente-Salar, NéstorKattelmann, KendraVieira-Potter, VictoriaKatuchova, JanaViennois, EmilieKatz, AbigailVierucci, FrancescoKaur, GunveenVignini, AriannaKawabata, KyuichiViljakainen, Heli T.Kawai, YoshichikaVilla-Bellosta, RicardoKawano, KouichiroVillani, AnthonyKayman, Kalantar-ZadehVillano, DeboraKazankov, KonstantinVillarini, AnnaKazlauskas, AndriusVillarreal, Jose TrevinoKeating, ElisaVilstrup, HendrikKeen, Douglas A. Vincent, GraceKeenan, MichaelVincent, John B.Keiler, Annekathrin MartinaVinceti, MarcoKeller, AmélieVinchi, FrancescaKeller, MarkusVinciguerra, ManlioKelley, DarshanVinson, JoeKello, MartinVioque, JesúsKellow, Nicole J.Virtanen, JyrkiKelly, AlanVisioli, FrancescoKennel, JulieVisser, AnitaKent, KatherineVita, RobertoKeshteli, Ammar HassanzadehVitale, MarilenaKessler, SonjaVitetta, LuisKhalesi, SamanVitturi, DarioKhare, SharadVlassopoulos, AntonisKharrazian, DatisVogel, ChristinaKhosla, PramodVolpato, MileneKhuder, Sadik A.Von Hundelshausen, PhillipKieliszek, MarekVon Hurst, PamelaKiely, MaireadVon Lintig, JohannesKienesberger, PetraVon Schacky, ClemensKim, Chang H.Von Wright, AtteKim, DongukVoortman, TrudyKim, HyounjuVormann, JürgenKim, JaehanVoznesenskaya, AnnaKim, Jae-wooWada, JunKim, Jung-AeWadehra, MadhuriKim, Ju-YoungWagner, AnikaKim, Kil-SooWahls, TerryKim, Mee ReeWainford, Richard D.Kim, Na-HyungWakabayashi, HidetakaKim, Ok-SuWaligora-Dupriet, Anne-JudithKim, Sung-HoonWalker, AlanKim, WeonWalker, ChristieKimura, TomokiWalker, D. CatherineKindler, JosephWalker, Marjorie. M.King, JanetWallace, Taylor C.Kinsky, SuzanneWalsh, Jennifer M.Kipp, AnnaWalton, Gemma EmilyKippler, MariaWalyaro, D. J.Kirk, BenWanders, DesireeKirkpatrick, SharonWang, BoKishida, TaroWang, Chi ChiuKishimoto, YoshimiWang, Chin-KunKitic, Cecilia MaryWang, DongKitissou, MarcelWang, Feng-ShengKlatzkin, Rebecca R.Wang, GuozhengKlaus, SusanneWang, LiangKleber, Marcus E.Wang, XinkunKling, Pamela J.Wang, YumingKłoda, KarolinaWang, ZhanweiKluger, Michael D.Ward, LeighKnapp, Caprice A.Wardenaar, Floris C.Kneepkens, C. M. FrankWargovich, MichaelKnight, AlissaWarr, David G.Knipping, KarenWasse, LucyKnoell, Daren L.Waterlander, WilmaKnol, JanWätjen, WimKnott, RachelWatkins, Adam J.Knudsen, Knud Erik BachWatkins, Colleen MarieKobayashi, HanakoWatkins, JuliaKoch, ManjaWatrowski, RafałKoehler, KarstenWatson, WilliamKoenigstorfer, JoergWatt, PeterKoh, Gar YeeWax, BenjaminKojima-Yuasa, AkikoWeaver, ConnieKok, DieuwertjeWeber, JanetKok, WouterWeber, LynnKolahdooz, FaribaWeghuber, DanielKolb, AndreasWei, GanKoleva, Petya T.Weickert, Martin O.Kones, RichardWeiergräber, MarcoKontny, UdoWeiler, HopeKoon, Hon WaiWeimann, ArvedKoot, Bart G. P.Weissgerber, Tracey L.Kopjar, MirelaWelham, SimonKoplin, JenniferWellard-Cole, LyndalKoppe, Janna G.Welsh, Jean A.Kornprat, PeterWelsh, MichaelKorosi, AnikoWelters, Hannah J.Kotchen, Theodore A.Wendin, KarinKouassivi Aglago, ElomWendler, ChristopherKouretas, DimitriosWeng, Han-RongKoutelidakis, AntoniosWentz, Laurel M.Koutoukidis, DimitriosWertz, Philip W.Koutsos, AthanasiosWesson, DonaldKovatcheva-Datchary, PetiaWest, DanielKoyama, YuWest, KeithKrähenbühl, StephanWestenbrink, SusanneKrau, Jeffrey A.Westerberg, Ane CecilieKrause, RolfdieterWhisner, Corrie M.Krebs, NancyWhite, John S.Kreider, RichardWhite, Sarah H.Krejpcio, ZbigniewWhitfield, PatriciaKrogfelt, KarinWhiting, SusanKruger, MarlenaWhybrow, StephenKrupa-Kozak, UrszulaWidmer, Hans RudolfKrupp, DanikaWierzejska, ReginaKu, SeockmoWieser, SimonKuang, ZhizhouWightman, Emma L.Kubena, KarenWilkinson, Jenny M.Kubicova, LenkaWillems, MarkKubis, Hans-PeterWilliams, Andrew R.Kubota, NaotoWilliams, Christopher S.Kuhlenschmidt, MarkWilliams, ElizabethKuk, Jennifer L.Williams, NancyKukreja, SubhashWilliams, Robert J.Kuligowski, MaciejWilliamson, GaryKulozik, UlrichWilliamson, KevinKumar, Jothi DineshWilson, PatrickKuno, ToshiyaWilson, TedKuo, Jen-MinWinham, DonnaKuo, Shiu-MingWinkler, Marion F.Kuo, TonyWirth, MichaelKurasaki, MasaakiWise, PaulKurrpa, KalleWitt-Enderby, PaulaKurz, TinoWitting, Paul K.Kusuda, SatoshiWittmann, GáborKuwabara, MasanariWoelwer-Rieck, UrsulaKuwahara, AtsukazuWolber, FranKuwahara, KeisukeWolfe, Robert R.Kuwana, TomomiWölnerhanssen, Bettina KarinKwak, Chung ShilWolters, MaikeKweon, Oh-KyeongWon, Kyu ChangKwiatkowska, KatarzynaWondrak, Georg T.Kwon, OranWong, Man-SauKyro, CecilieWong, RachelKyrozis, AndreasWoo, Jessica GrausLa Favor, Justin D.Worsley, AnthonyLa Frano, Michael R.Woźniak, ŁukaszLa Vieille, SébastienWright, MeleciaLabruna, GiuseppeWu, Chi-ReiLac, AndrewWu, FeitongLachenmeier, DirkWu, Li-ChenLacy, KatieWu, Tzu-HuaLagouri, VasilikiWu, Wen-TzuLaguna, Joan-CarlesWulf, Hans ChristianLaguzzi, FedericaWullaert, AndyLahiri, AmitWunderlich, Shahla M.Lai, I-JuWydau, SandraLai, Jun S.Xavier, Cristina Pinto RibeiroLaird, EamonXiaoli, AlusLaitinen, KirsiXie, GuoxiangLalas, StavrosXinyin, JiangLam, Yan Y.Xiong, ZhaohuiLamacchia, CarmelaXu, Feng-LianLammi, CarmenXu, HangLamousé-Smith, Esi S. N.Xu, JingLamuela-Raventós, RosaXue, XiangLand, Mary-AnneXun, PengchengLandberg, Rikard Yabe, DaisukeLandete, José MariaYamada, YosukeLandis-Piwowar, KristinYamaguchi, YasuhiroLandrier, Jean-françoisYamaki, KohjiLane, DariusYamamoto, TakayukiLangouche, LiesYamanaka-Okumura, HisamiLangsetmo, Lisa A.Yamazaki, EtsukoLanou, Amy JoyYamazaki, TomomiLaparra, Jose MoisésYan, BowenLarsen, Bodil M. K.Yang, GongLasekan, John B.Yang, Hsin-YiLass, AchimYang, Jae WookLatella, GiovanniYang, QiyuanLaughlin, MarenYang, Ray-YuLauritzen, LotteYang, Rong-SenLavender, AndrewYang, Woong-MoLaverty, Anthony A.Yang, YueLavoie, Jean-claudeYannicelli, StevenLawen, AlfonsYano, ShozoLawrence, Catherine B.Yaqoob, ParveenLawrence, KarlYarmolinsky, LudmilaLayman, DonaldYasuda, KazukiLe Couteur, David G. Yatabe, JunichiLê, Kim-AnneYatera, KazuhiroLeach, Steven T.Yeates, AlisonLecerf, Jean-michelYee, Jennifer K.Lecoultre, VirgileYeh, Kuo-WeiLedda, CaterinaYeh, Shu-LanLee, Hyeon YongYeh, Sung-LingLee, JasonYi, StellaLee, Jun HeeYian, GuLee, JunsooYiannakopoulou, EugeniaLee, Karen Chia-LunYim, Jung-EunLee, Kwang HoYimam, MesfinLee, Min WonYoder, MervinLee, Ming-FenYohei, ShirakamiLee, Ok-HwanYokota, TomoyaLee, Sang GilYolton, KimberlyLee, Sang-HanYoneyama, TadakatsuLee, Seung-YeonYoshida, TadashiLee, Sun EunYoshihito, YokoyamaLee, Sun YoungYoshinaga, JunLee, Sun-OkYoshino, JunLee, Tzong-ShyuanYoshiyama, HironoriLee, Vincent WsYoung, AntonyLee, Yeon-JuYu, Chia-LiLefevre, MichaelYu, SiwangLeffler, JonatanYu, ZhipingLegeza, BalázsYuan, LijuanLehtisalo, JenniYui, KunioLeite, João CostaZaborskis, ApolinarasLemieux, SimoneZafeiridis, AndreasLenzen, SigurdZakrzewski-Fruer, JuliaLeonard, Maureen M.Zamora-Ros, RaulLephart, Edwin D.Zand, NazaninLeri, FrancescoZang, LiqingLessard, LauraZanini, BarbaraLeung, Chung-HangZanon-Moreno, VicenteLeung, Ricky Yuet-KinZanotti, IlariaLevenson, AnaitZarogoulidis, PaulLevinger, PazitZastre, JasonLevinson, Ralph D.Zavacki, Ann-MarieLevy, LouisZavattari, PatriziaLewinska, AnnaZdunczyk, ZenonLewis, Nathan A.Zeinstra, GertrudeLewis, RohanZeman, Daniel MeierLeyse-Wallace, RuthZemel, MichaelLi, ChunZeng, HuaweiLi, DuoZevallos, Victor F.Li, Jau-YiZgaga, LinaLi, LiZhang, BingLi, QianjinZhang, DeqiangLi, WeijuanZhang, HuaLi, Wen-WuZhang, JianLi, YanZhang, JianjunLiao, Jyh-feiZhang, LipingLiao, Yi-JenZhang, YumeiLiaset, BjørnZhao, LiangLiberato, Selma C.Zheng, JoleneLidon, FernandoZheng, M.Lieben, CindyZheng, WenxinLiem, GieZhou, JingyingLiguori, MariaZhu, ChrisLilenfeld, LisaZhu, Fengqing MaggieLim, JunxianZhu, HuaLim, KarenZhu, ZongjianLin, Chih-ChienZhuang, ShougangLin, Chih-LiZiegler, EkhardLin, DanielZiegler, JaneLin, Hui-HsuanZilversmit, LeahLin, Pao-HwaZimmermann, PetraLin, Pei-HuiZinman, BernardLin, Ping-TingZittermann, ArminLin, Shih-HuaZivkovic, AngelaLin, Shyh-HsiangZlatko, NikoloskiLionetti, VincenzoZmijewski, MichalLiou, Ying-mingZock, Peter L.Lira, FabioZosky, GraemeLittle, Tanya J.Zou, PingLiu, JianZuccotti, Gian V.Liu, Pei-YangZuhl, MicahLiu, Shing-HwaZwilling, ChrisLiu, Yang


